# An Interesting Prenatal Diagnosis: Double Aneuploidy

**DOI:** 10.1155/2013/790286

**Published:** 2013-12-04

**Authors:** Çetin Aydin, Serenat Eris, Yakup Yalcin, Halime Sen Selim

**Affiliations:** ^1^Gynecology and Obstetrics Department of Ataturk Training and Research Hospital, Basin Sitesi, Yesilyurt, 35360 Izmir, Turkey; ^2^Gynecology and Obstetrics Department of Van Training and Research Hospital, Süphan Street, 65300 Van, Turkey

## Abstract

Double aneuploidy, the existence of two chromosomal abnormalities in the same individual, is a rare condition. Early diagnosis of this condition is important to offer termination of pregnancy in genetic counselling. Cytogenetic analysis with amniocentesis and ultrasound examination is valuable for diagnosis of double aneuploidy. In this report we present a case with the karyotype of 48XXY+21 diagnosed prenatally.

## 1. Introduction

Since prenatal diagnosis with cytogenetic analysis has been introduced, the detection of chromosomal abnormalities has become possible. Chromosomal abnormalities occur in 0.1% to 0.2% of live births and the most common aneuploidy among live born infants is Down' syndrome [1].

The first case of double aneuploidy with trisomy 21+xxy was reported in 1959 by Ford et al. [2]. Several cases with various combinations of double aneuploidy have been found since the first report. Most reported cases of double aneuploidy are presented in the form of spontaneous abortions [3]. The coincidence in the same individual of both Down's and Klinefelter's syndromes seems to be a relatively rare condition and its clinical presentations are variable. The manifestations of both chromosomal abnormalities can be found depending on the predominating aneuploidy or a combination effect of both [4, 5].

In this report we present a case with the karyotype of 48XXY+21 diagnosed prenatally.

## 2. Case Presentation

A 19-year-old nulliparous woman was referred for counselling and amniocentesis at 17-week gestation after a first trimester maternal combined screening carried out at 12 weeks showed a high risk for DS. Nuchal translucency measurement was 5.2 mm (2.98 MoM) at 12-week and 4-day gestation. Multiples of medians (MoMs) for PAPP-A and free B-HCG were determined to be 0.41 and 0.99, respectively. While the age-related risk was only 1 : 1500, the calculated Down' syndrome risk was 1 : 10.

The paternal age was 20 years. Parents were an unrelated couple married for 6 months, who had not had unusual X-ray exposure, diabetes, or thyroid disease. The parental karyotypes were found to be normal.

**Figure 1:**
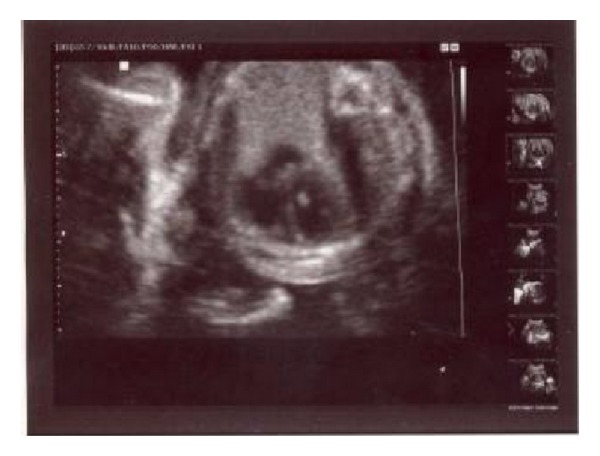
Echogenic cardiac focus in left ventricle.

**Figure 2:**
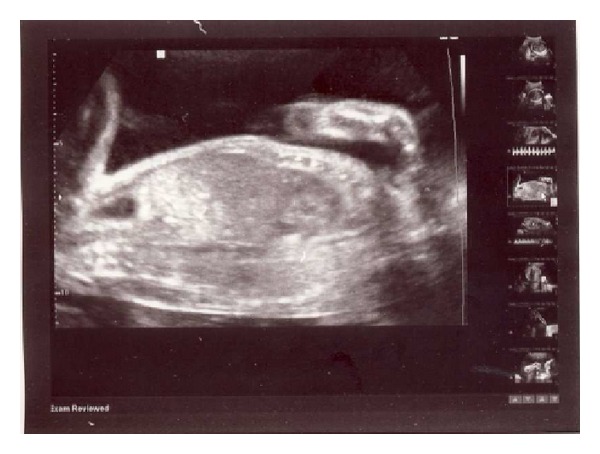
Echogenic bowel.

Ultrasound examination revealed a single fetus and marked polyhydramnios. Echogenic intracardiac focus in left ventricle and echogenic bowel were also detected (Figures 1 and 2). Fetal biometry showed a biparietal diameter of 55 mm (22 weeks 6 days) and femur length of 41 mm (23 weeks and 2 day).

**Figure 3:**
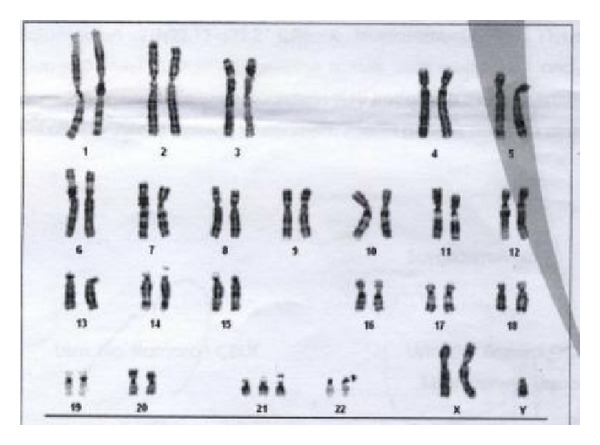
Karyotype of the fetus with 48XXY+21.

Extensive counselling for the unrelated couple was made and ultrasound-guided transamniotic amniocentesis was carried out through a single uterine puncture with a 21 G needle. Results of the chromosomal culture from amniocentesis showed an abnormal fetal karyotype of 48XXY+21 (Figure 3). For congenital problems usually 20 metaphase cells are scored. To rule out mosaicism we scored 50 metaphase cells.

The patient was recalled for genetic counselling and they preferred termination of pregnancy.

Medically induced termination of pregnancy was carried out without complications. The gestational age at the time of induced delivery was 23 weeks. A male infant weighting 680 gr was delivered. He died forty-five minutes after delivery.

Parental consent was obtained before autopsy. On postmortem examination of the neonate, immature internal organs and hyponasal bridge were detected. Echogenic bowel and echogenic intracardiac focus could not be confirmed at autopsy.

## 3. Discussion

Various methods such as biochemical markers, amniocentesis, and prenatal ultrasound have been used to identify women at risk of carrying fetus with chromosomal abnormalities [6]. Double aneuploidy, the existence of two chromosomal abnormalities in the same individual, is a rare condition.

It has been generally thought that patients with one chromosomal abnormality are more likely to have a second chromosome abnormality [4]. Harnden et al. (1960) have reported that coincidence of DS and KS in the same individual might be expected in 1/560000 birth [7].

We report here a case of a neonate who exhibited karyotype 48XXY+21 with increased nuchal translucency measurement and ultrasound findings such as echogenic intracardiac focus, echogenic bowel, and polyhydramnios.

Several studies have reported that increased fetal NT is associated with fetal abnormalities such as cardiac defects, diaphragmatic defect, exomphalos, skeletal defects, and genetic disorders. With fetal NT above 3.5 mm as in our case, a fetal karyotyping should be offered [8].

Findings in fetuses with sonographically detectable aneuploidies include both structural abnormalities and nonstructural abnormalities or markers. Soft markers may be seen in normal population but have an increased incidence in fetus with chromosomal abnormalities. The most commonly studied soft markers are a thickened nuchal fold, choroid plexus cyst, echogenic intracardiac focus, echogenic bowel, renal pyelectasis, and femoral shortening [9].

Fetal echogenic bowel is the presence of hyperechoic bowel at sagittal image of fetal abdomen. Several studies have been published about the association of echogenic bowel with aneuploidy. Although it can be found 0.5% in normal fetuses, a detailed investigation should be performed. It is known to be observed 6-7 times higher in fetuses with aneuploidy [10].

Echogenic intracardiac foci are echogenic areas in the region of papillary muscle in either cardiac ventricle at four chamber view. Echogenic intracardiac focus is observed in 3-4% of normal fetuses but it is related to increased risk in aneuploidy [11]. Although results suggest poor accuracy for ultrasonographic soft markers, it is common practice for clinicians to use them as evidence of an increased risk of aneuploidy.

Szigeti et al. have reported that when correlation of prenatal sonographic diagnosis and morphologic findings of fetal autopsy in fetuses with aneuploidy examined, 2 soft markers seem to have limited value: echogenic bowel cases and cases with echogenic intracardiac focus could not be confirmed at autopsy similar to our case. Aneuploidy is associated with abnormal bowel function in newborns. It is hypothesized that a similar process in the fetus causes echogenic bowel. So echogenic bowel is a sonographic feature and autopsy is not able to evaluate these signs [12].

A pathologic study by Roberts and Genest in 1992 [13] found papillary muscle calcification in 16.5% of fetuses with Trisomy 21 and 38.9% of fetuses with Trisomy 13. We could not verify prenatal ultrasound findings of echogenic intracardiac focus postnatally. It could be due to the loss of tissue during the serial of the formalin-fixed, paraffin-embedded samples. However, a potential for misdiagnosis due to specular reflections within the fetal heart, producing echoes to be mistaken for EIF, can not be ruled out.

In patients with Down-Klinefelter syndrome, the Down syndrome phenotype often predominates. Characteristic features of Klinefelter's syndrome do not occur until puberty.

Table 1 lists the available sonographic findings of the patients with a prenatal diagnosis of 48XXY+21. According to these cases, prenatal diagnosis relies on sonographic markers for Down's syndrome rather than Klinefelter's syndrome. In published cases prenatal diagnosis of Klinefelter's syndrome seems to be incidental as a result of amniocentesis performed for increased risk for DS similar to our case [14].

It is known that the most common cause of aneuploidy is meiotic nondisjunction [15].

The cause of nondisjunction is uncertain. The most favored explanation is advanced maternal age [4, 16]. In our case the neonate was delivered by a teenage mother, reversely.

In conclusion early diagnosis of this condition is important to offer termination of pregnancy in genetic counselling. This report will be helpful to better understand the phenotype-genotype relationship of double aneuploidy with trisomy 21+XXY.

## Figures and Tables

**Table 1:** Prenatally diagnosed cases of 48 XXY+21.

Study	Sonographic findings
Sanz-Cortés et al. [17]	Tridigital syndactylia in left hand, low set ears

Moog et al. [18]	Hygroma, thoracic skin edema, and small heart

Metzenbauer et al. [19]	Normal NT of 2 mm

Smith et al. [20]	Clinodactyly, bilateral brachymesophalangia of the 5th digit

Glass et al. [21]	None

Jeanty and Turner [14]	Absent nasal bone, bilateral brachymesophalangia of the 5th digit, short femur, and humerus

Our case	Echogenic intracardiac focus in left ventricle, echogenic bowel, and polyhydramnios
